# Characterizing tuberculous meningitis in a South African pediatric cohort using GCxGC-TOFMS metabolomics

**DOI:** 10.1007/s00430-025-00857-9

**Published:** 2025-09-27

**Authors:** Anouska Mangaroo-Pillay, Du Toit Loots, Regan Solomons, Shayne Mason

**Affiliations:** 1 Human Metabolomics, Faculty of Natural and Agricultural Sciences, North-West University, Potchefstroom, South Africa; 2 Department of Paediatrics and Child Health, Faculty of Medicine and Health Sciences, Stellenbosch University, Cape Town, South Africa

**Keywords:** Tuberculous meningitis (TBM), Cerebrospinal fluid (CSF), Two-dimensional gas chromatography - time-of-flight mass spectrometry (GCxGC-TOFMS), Metabolomics, Metabolic characterization, Neurometabolism

## Abstract

The objective of this study was to characterize the metabolic profile of tuberculous meningitis (TBM) in the cerebrospinal fluid (CSF) of a South African pediatric cohort using two-dimensional gas chromatography linked to time-of-flight mass spectrometry (GCxGC-TOFMS). A metabolite extraction and derivatization protocol was performed using CSF samples from 21 cases of bacteriologically confirmed TBM and 24 controls without meningitis, and analyzed by GCxGC-TOFMS, followed by univariate and multivariate statistical comparisons to identify differentiating compounds. A total of 48 metabolites (involved in glycolysis and amino acid and fatty acid metabolism) were identified that differentiated the two groups (effect size *d*-value > 0.5 and partial least squares discriminant analysis VIP value > 1.0, with 16 metabolites highlighted as significant (*p*-value < 0.05). Eight novel metabolites (linked to imines, alkenes, and volatile organic compounds), never before identified in a TBM study, characterize TBM in this cohort. This study confirms existing metabolomics research aimed at characterizing the metabolic profile of TBM. Some affected metabolic pathways include glycolysis, pentose phosphate pathway, amino acids, and fatty acid metabolism. New information is provided on the effect TBM has on neurometabolism, how TBM can reduce the neuroprotective function of some metabolites in the brain, resulting in the neurological symptoms typically associated with this disease, and contribute to our understanding of the pathogenesis of TBM; ultimately, contributing to the development of a metabolic model of TBM.

**Supplementary Information:**

The online version contains supplementary material available at 10.1007/s00430-025-00857-9.

## Introduction

Tuberculous meningitis (TBM), a severe form of extrapulmonary tuberculosis (TB), is caused by an infection of the brain by *Mycobacterium tuberculosis* (*M.tb*) [1]. The pathogenesis of TBM is complex, and much remains unknown. However, what is known is that *M.tb* bacilli are transmitted by inhaled airborne droplets and primarily infect alveolar macrophages in the lungs [2]. Thereafter, the bacilli enter the vasculature, followed by the central nervous system (CNS) by traversing the blood–brain barrier (BBB). Once in the brain, the immune host response to the infecting *M.tb* bacilli results in lesions that become surrounded by immune cells to form granulomas in the meninges and adjacent brain parenchyma [2]. Cerebrospinal fluid (CSF) circulates throughout the brain and is the ideal biofluid for the diagnosis of TBM.

The metabolome of the CSF is directly indicative of the metabolite production/changes that occur in the brain and thus serves as an ideal matrix for investigating changes in the CNS induced by various CNS diseases, such as TBM [3]. Current diagnostic CSF biochemical indicators of TBM are low glucose levels, elevated protein levels, and elevated opening pressure [4]. The ‘gold standard’ for diagnosing TBM involves the identification of *M.tb* bacilli by microscopy, culture, and nucleic acid amplification in the CSF [1, 2, 5]. However, the *M.tb* load is usually low in CSF, typically resulting in inconclusive diagnostic results. One of the challenges with these diagnostic tests is that 6–10 mL of CSF is required, which is particularly difficult to obtain in children and takes several weeks. The delayed diagnosis of TBM subsequently results in delayed treatment and an increase in morbidity and mortality. Hence, there is a need for the identification of additional biomarkers and assays to aid in better characterization of this disease, and faster and more efficient differential diagnosis of TBM.

Metabolic characterization is the first step towards the identification of novel metabolic biomarkers. Existing literature highlights the use of ^1^H-NMR spectroscopy as an analytical platform to investigate TBM [6, 7]. The main limitation of ^1^H-NMR, compared to mass spectrometry, is lower sensitivity; hence, fewer differentiating metabolites are identified [8]. Two-dimensional gas chromatography linked to time-of-flight mass spectrometry (GCxGC-TOFMS) allows for the analysis of lower-abundant metabolites that are usually not detectable by ^1^H-NMR. Moreover, to the best of our knowledge, GCxGC-TOFMS has never been used to analyze CSF collected from pediatric patients with TBM. The aim of this study was to use GCxGC-TOFMS as an analytical platform for the metabolic characterization of CSF samples collected from a South African pediatric TBM cohort.

## Methods

### Sample collection and ethics

The samples used in this study were from a retrospectively collected sample cohort (2009–2018) from a larger study. As part of the larger study, the samples were collected at Tygerberg Hospital in the Western Cape Province of South Africa, a tertiary academic hospital serving the Eastern Metro of Cape Town and a large part of the Western Cape Province of South Africa, a TB-endemic area. All participants in this study were children (aged 3 months to 12 years) suspected of having meningitis. The control group in this study was confirmed negative for any form of meningitis (Non-Meningitis). The control group was divided into two sub-groups, where one sub-group did not show neurological symptoms, referred to as the Non-Neuro Non-Meningitis group (n = 24), and the other sub-group (Neuro Non-Meningitis; n = 31) had cases that were meningitis negative but presented with neurological symptoms. The experimental group consisted of bacteriologically-confirmed (definite) TBM (Def TBM; n = 21), diagnosed based upon the uniform research case definition of TBM by Marais et al. [9], and probable TBM (Prob TBM; n = 31), where CSF was smear negative for AFB and culture negative for *M.tb* but presented with clinical symptoms suspected to be TBM, and one or more of the following four criteria were met: chest X-ray consistent with pulmonary tuberculosis; other specimens (sputum, lymph node, gastric washings) positive for AFB; evidence of extrapulmonary tuberculosis; and brain imaging (CT or MRI) evidence of TBM. All CSF samples collected for research purposes were excess volume after the differential diagnosis of pediatric patients was performed. The Def TBM and Non-Neuro Non-Meningitis groups were compared in the primary analysis of this study to generate a metabolic model, and the Prob TBM and Neuro Non-Meningitis groups were used to test the robustness of this metabolic model. Written and informed consent and/or assent were obtained from each participant. This study was approved by the North-West University (NWU) Human Research Ethics Committee (approval number NWU-00063-18-A1-03), as well as the Stellenbosch University Human Research Ethics Committee (approval number N16/11/142). HIV-positive/unknown cases were excluded from this study because HIV co-infection confounds the already complex CSF metabolic profile. A summary of demographic and clinical information for the Def TBM and Non-Neuro Non-Meningitis groups can be found in Table 1.

**Table 1:** Summary of demographic and clinical information of the Def TBM and Non-Neuro Non-Meningitis groups

	Non-neuro non-meningitis (N = 24)	Def TBM (N = 21)
Age in months		
Median [IQR]	25.5 [11.5–69]	44 [28–78]
TBM stage on admission (n [%])		
Stage 1	N/A	6 [29]
Stage 2a	N/A	3 [14]
Stage 2b	N/A	5 [24]
Stage 3	N/A	7 [33]
Concurrent pulmonary TB (n [%])	0	5 [24]
Concurrent miliary TB (n [%])	0	7 [33]
CSF cell count (cells per µL CSF)		
Erythrocytes (median [IQR])	0 [0–26]	28 [6–155]
Leukocytes (median [IQR])	1 [0–2]	185 [69–265]
PMNs (median [IQR])	0 [0]	11 [3–28]
Lymphocytes (median [IQR])	1 [0–2]	160 [64–237.25]
CSF protein (g/L) (median [IQR])	0.21 [0.17–0.3]	1.53 [1, 2]
CSF glucose (mmol/L) (median [IQR])	3.8 [3.3–4.15]	1.5 [1.15–3.25]

IQR, interquartile range; PMN, polymorphonuclear neutrophils

### Experimental design

**Fig. 1:**
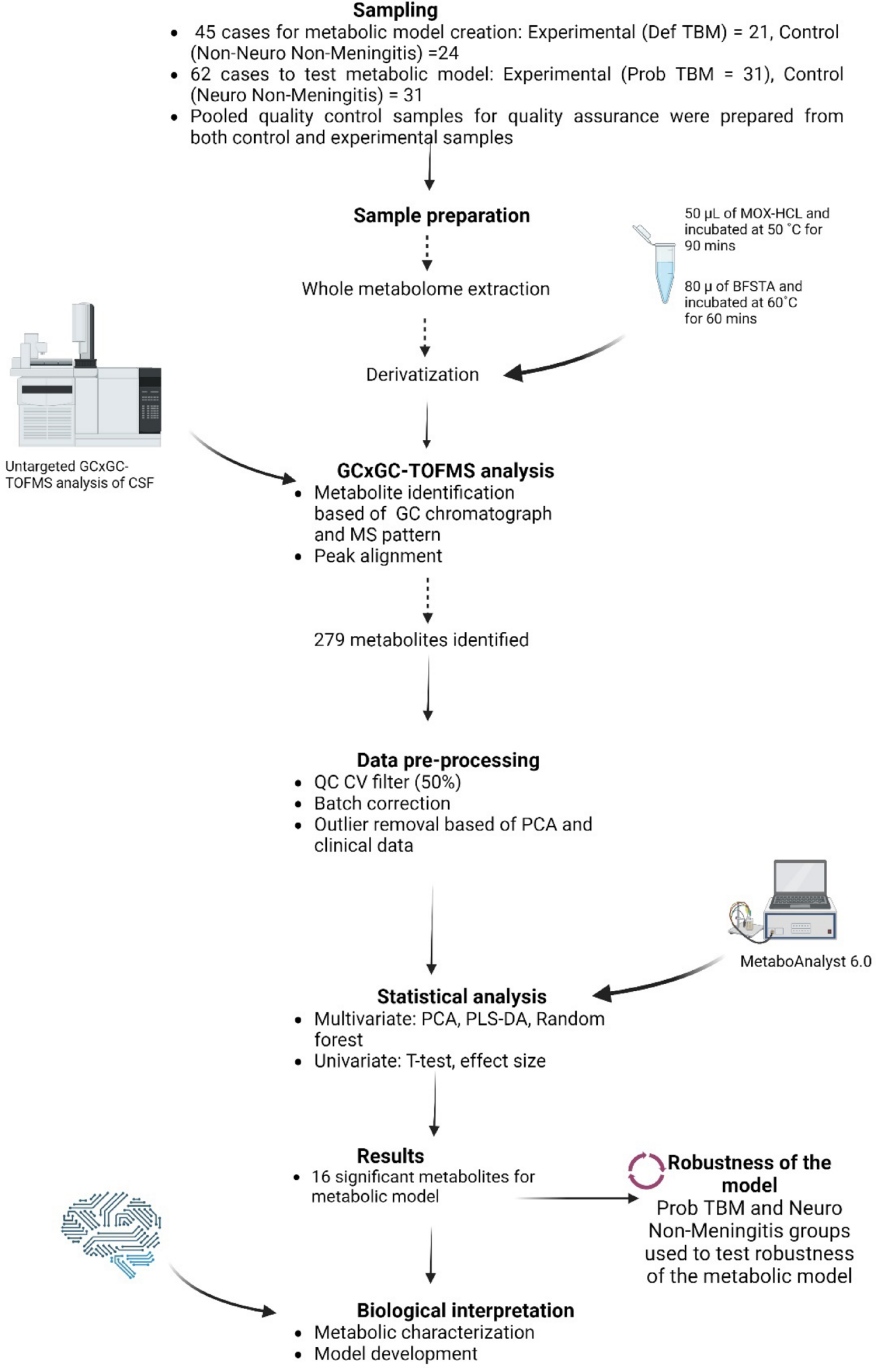
Schematic of the experimental design—encompassing the sample selection, preparation, and analysis, statistical analysis, results, and biological interpretation

Figure 1 illustrates the experimental design of this study, including: sampling, sample preparation, analytical analysis, data pre-processing, statistical analyses, testing the robustness of the metabolic model, and biological interpretation of the results. The preparation of the samples involved a whole metabolome extraction and a two-step derivatization; thereafter, samples were analyzed in a randomized injection sequence order on a GCxGC-TOFMS, all according to established protocols. A pooled quality control sample (QC; created by pooling a set volume of CSF from each sample) was used for quality assurance purposes. For data processing, raw data were sorted and cleaned using the LECO ChromaTOF software (version 4.32). QCs were checked for quality assurance, and batch correction was applied. Outlier detection and removal were performed. Statistical analysis was done using MetaboAnalyst 6.0. Multivariate statistics were done on log-transformed and auto-scaled data. The comparison of the Def TBM and Non-Neuro Non-Meningitis groups was done to create a metabolic model for TBM, and the Prob TBM and Neuro Non-Meningitis groups were used to test the robustness of this metabolic model. Finally, a biological interpretation was done whereby the significant metabolites were examined and the metabolic pathways were elucidated to describe TBM.

### Reagents

An internal standard (IS) solution was prepared by weighing 12.5 mg of 3-phenylbutyric acid and adding 25 mL of methanol (50 ppm). The metabolite extraction solution consisted of a 1:2:1 ratio of chloroform: methanol: water, respectively. The chloroform and methanol used were obtained from Honeywell International Inc. (Muskegon, Michigan, USA). The derivatizing reagents included N,O-bis(trimethylsilyl)trifluoroacetamide (BSTFA) with 1% trimethylsilylchloride (TMSC) and a 15 mg/mL solution of methoxamine hydrochloride (MOX-HCl) in pyridine, both obtained from Sigma-Aldrich (St. Louis, Missouri, USA).

### Sample preparation

The samples were prepared by whole metabolome extraction, as previously described by Luies and Loots [10]. Briefly, 50 µL of the prepared IS was added to 100 µL of the sample. Next, 500 µL of the prepared metabolite extraction solution was added and dried under a nitrogen stream for 45 min in a heating block set at 40 °C. Thereafter, 50 µL of MOX-HCl was added to each sample, briefly vortexed, and incubated at 50 °C for 90 min. Lastly, 80 µL of BFSTA was added, and the samples were incubated at 60 °C for 60 min.

### GCxGC-TOFMS analysis

All derivatized samples (and QCs) were loaded onto the autosampler, and 1 µL was injected in a 1:5 split ratio for analysis using a Pegasus 4D GCxGC-TOFMS, comprising an Agilent 7890A gas chromatograph (Agilent, Atlanta, USA) and a TOFMS from LECO Africa (LECO Africa (Pty) Ltd, Johannesburg, South Africa). The instrument specifications were identical to those of Luies and Loots [10]. The chromatograph consisted of a capillary column, with dimensions 19.90 m length × 180 µm internal diameter × 0.18 µm film thickness, at a maximum temperature of 325 °C. The carrier gas was helium, which flowed constantly at 1 mL/min. For the primary column, the initial temperature was held at 250 °C for 1 min, then increased to 300 °C for 2 min at a rate of 20 °C/min. The transfer line temperature was set to 225 °C, with the ion source temperature at a constant 200 °C. For the TOFMS, the acquisition delay was set at 350 s, and an acquisition rate of 20 spectra/sec. The detector voltage was 50 V, and the mass spectra were collected over a range of 50–800 m/z at an acquisition rate of 20 spectra/sec. The data were processed using the LECO ChromaTOF software (version 4.32).

### Data pre-processing

For the spectral match, the mass threshold was 10, with the minimum similarity match being 700. The retention time match criteria were set at 1 for the maximum number of modulation periods apart and 0 s for the maximum retention time differences. Peak alignment and identification were performed at a signal-to-noise (S/N) ratio of 20. To define the analytes to keep, the minimum number of samples that contain the analyte was set at 5, and the minimum percent of samples in a class containing the specified analyte was set at 50. Lastly, the peaks were identified by comparing their signature mass fragmentation patterns and retention times against existing libraries. Additional data processing was done according to in-house procedures modelled after Luies and Loots [10]. Briefly, artefactual compounds from the derivatization process were removed from the dataset, and multiple identities were merged based on retention times and compound masses. Subsequently, each compound was normalized relative to the IS, and a zero filter (> 50%) was applied to the data set. Using only the quality control (QC) samples, the batch effect was determined and corrected. After batch correction, a CV filter was applied (> 60%) to the sample set to remove unreliable variables. On visual inspection of the principal component analysis (PCA) generated from the data set, outlier removal was completed. The final data set was then generated for statistical analysis.

### Statistical analysis

Both univariate (t-test, fold change, volcano plot, and effect size) and multivariate (PCA, partial least squares discriminant analysis (PLSDA), and random forest) statistics were applied. PCA was used at three points during data evaluation to: 1) check the QCs to determine batch effect; 2) identify outliers in the two experimental groups; and 3) qualitatively evaluate the natural differentiation between the experimental and control groups. For PLSDA, a VIP value greater than 1.0 for component 1 and component 2 was used to quantitatively identify metabolites of importance. A random forest model was used to support the PLSDA results, as well as to evaluate the classification of the multivariate metabolic model. The permutation *p*-value of the PLSDA and the out-of-the-box value of the random forest model were checked for significance (< 0.05). For the univariate measures, a t-test with an FDR *p*-value < 0.05 was considered statistically significant, and a Cohen’s effect size d-value > 0.5 and fold change value > 1.5 were considered for practical significance. All statistical analyses were done using MetaboAnalyst 6.0.

## Results

### Data pre-processing and quality assurance

Using only QC samples, a batch effect was determined, and a manual batch correction was performed using quantile equating, where quantile equating aims to adjust the data for differences across the data set [11]. A subsequent PCA (see Fig. S1) was done on the batch-corrected data, and three Non-Neuro Non-Meningitis samples were found lying outside the 95% confidence interval ellipsis and flagged as outliers. Upon reviewing the clinical data of these three outlier samples, they were retained in the data set because there was no clinical reason for exclusion. In addition to these three outlier samples, five Non-Neuro Non-Meningitis samples were found to overlap with the TBM group (see Fig. S1). One of these five samples had clinical support to be removed (the patient had a chest X-ray that indicated pulmonary TB and was receiving TB medication); however, the other four samples did not show clinical reasons for exclusion and were retained. Hence, only one Non-Neuro Non-Meningitis sample was removed as an outlier, and the final number of samples evaluated per group was 21 for the Def TBM group and 24 for the Non-Neuro Non-Meningitis group. A total of 279 metabolites were identified for statistical analyses.

### Qualitative evaluation of data

**Fig. 2:**
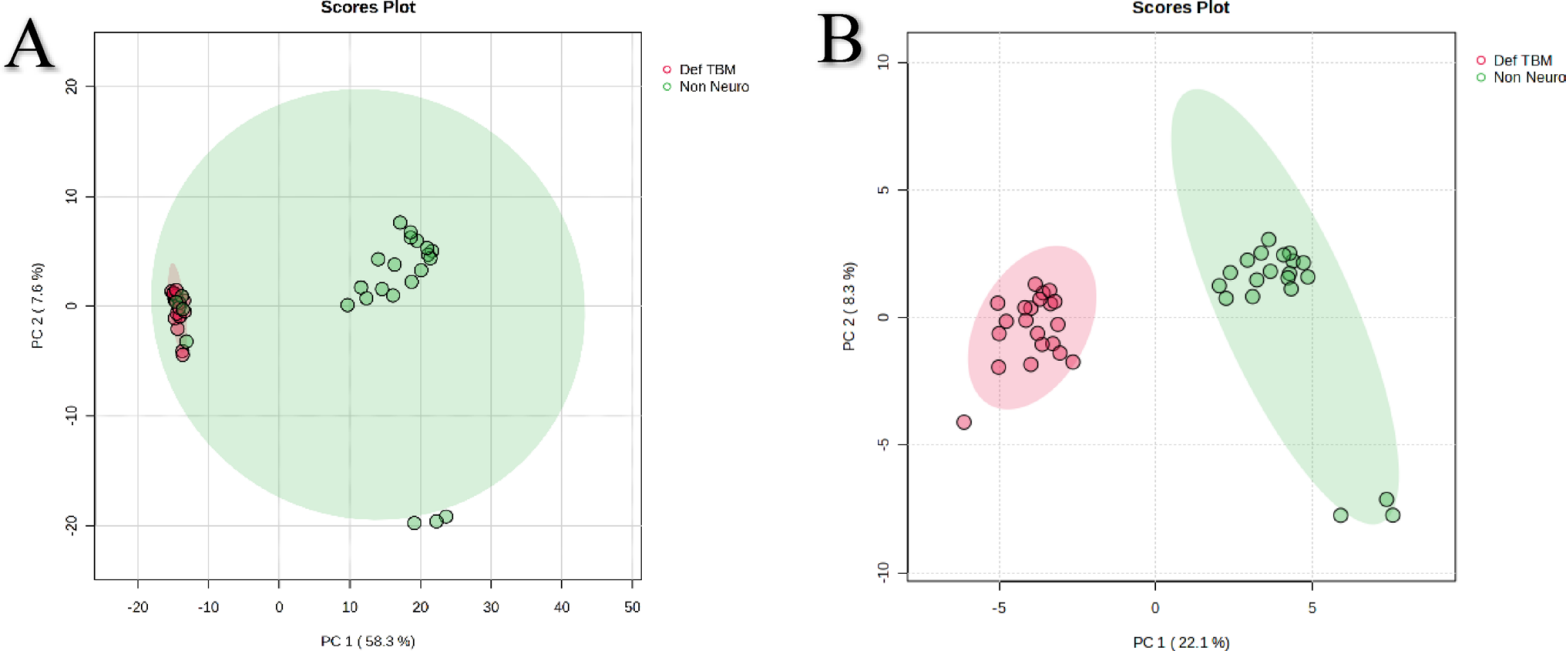
PCA scores plot of PCA 1 versus PCA 2 of the definite TBM [Def TBM (n = 21); red] and control [Non-Neuro Non-Meningitis (n = 24); green] groups. **A** The PCA shows the two groups after one outlier sample was removed, which had clinical reasons for removal (clinical symptoms and a high level of medication). The four Non-Neuro Non-Meningitis samples that overlap with the TBM group remain because there is no valid reason to remove them. Three Non-Neuro Non-Meningitis samples lie just outside the 95% confidence interval ellipsis, but they also did not have a clinical reason to be removed. The overall variation explained by this PCA scores plot is 65.9%. **B** The PCA shows complete natural separation between the two groups after removing 4 Non-Neuro Non-Meningitis samples overlapping with the TBM group. The overall variation explained by this PCA scores plot is 30.4%

Following outlier removal, another PCA was performed (Fig. 2A) to qualitatively evaluate the clustering within each group and the natural differentiation between the Def TBM and Non-Neuro Non-Meningitis groups. It can be seen from Fig. 2A that the TBM samples cluster tightly together into a homogenous group. In contrast, the Non-Neuro Non-Meningitis group has several samples that cluster homogenously near the center of the scores plot. However, three samples from the Non-Neuro Non-Meningitis group lie just outside the 95% confidence interval ellipses, and four Non-Neuro Non-Meningitis samples overlap with the Def TBM samples. As an exercise, these four Non-Neuro Non-Meningitis cases overlapping with the TBM group were removed, and the PCA analysis was re-done (Fig. 2B), which yielded complete natural separation between the two groups. At this point, it must be noted that we performed the quantitative statistical analyses (described below) on both Fig. 2A, B, and the results yielded the same list of important metabolites responsible for the differentiation between the two groups. However, since the PCA scores plot in Fig. 2A explains 65.9% of the total variation in the data (versus the 30.4% total variation explained by the PCA in Fig. 2B), it was decided to retain Fig. 2A and continue with the quantitative statistical analyses using the data from Fig. 2A (i.e., the four Non-Neuro Non-Meningitis samples overlapping with the TBM group were not removed from the subsequent quantitative statistical analyses).

### Quantitative statistical analysis

**Fig. 3:**
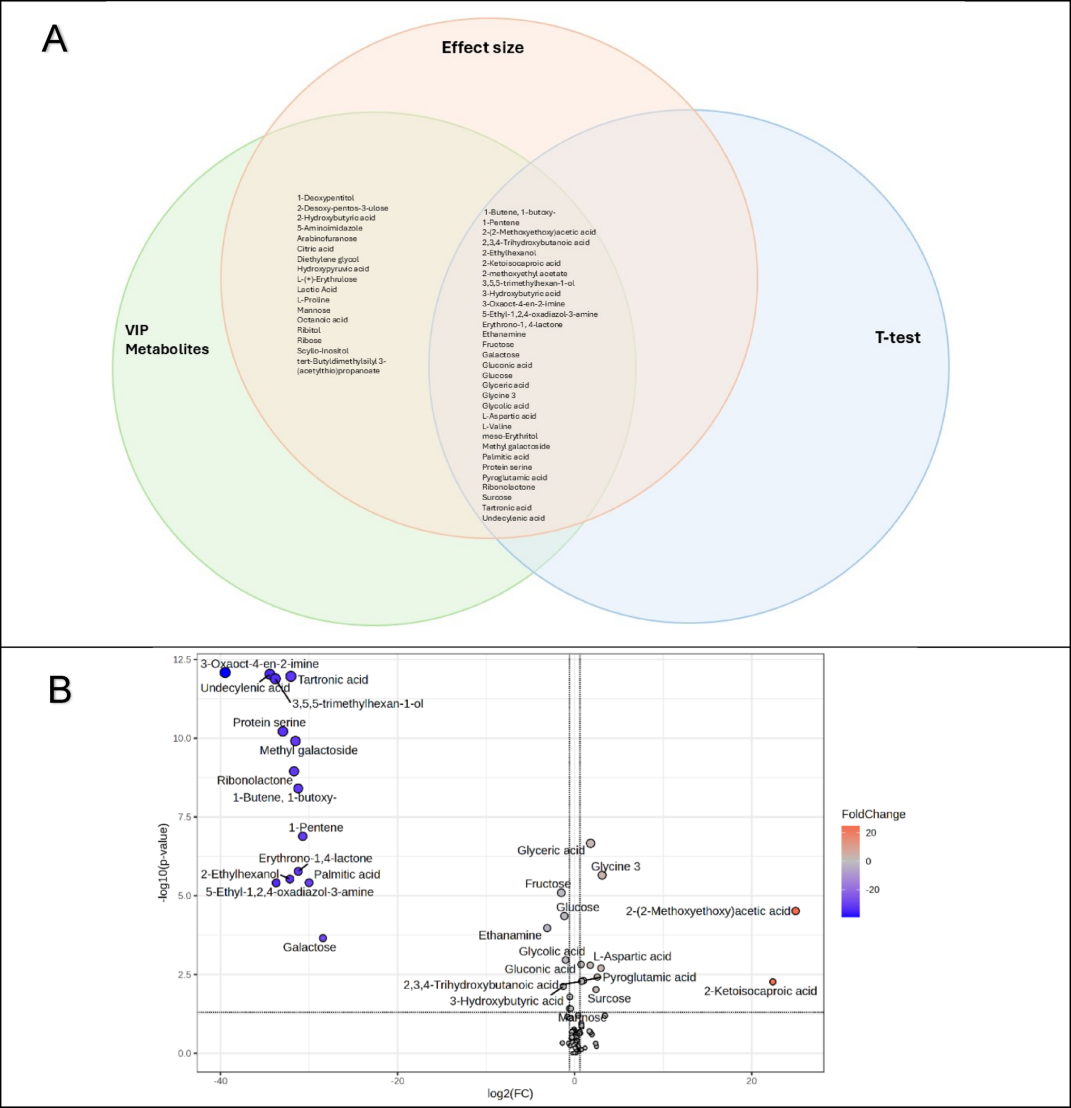
Quantitative statistical tests performed on the Def TBM and Non-Neuro Non-Meningitis groups, where **A** reflects a Venn diagram containing the list of metabolites identified for each test (FDR *p*-value < 0.05, effect size > 0.5, and VIP > 1.0). A total of 31 metabolites meet at least one of these cut-off criteria, 15 metabolites have a *d*-value > 0.5 and VIP > 1.0 but do not have a *p*-value < 0.05, and 16 metabolites meet all three criteria—VIP metabolites. **B** Volcano plot showing the fold change (log-transformed with base 2) with a threshold of 1.5 on the x-axis and the t-test *p*-value (log-transformed with base 10) with a threshold of 0.05 on the y-axis. The metabolites identified above the thresholds are important based on univariate statistics for differentiating the Def TBM group from the Non-Neuro Non-Meningitis group. The blue dots on the left indicate metabolites significantly decreased in the TBM group, and the red dots on the right indicate significantly increased metabolites in the TBM group

Based on the PLSDA (R^2^ = 0.85; Q^2^ = 0.67) (see Fig. S2), 26 metabolites had VIP values > 1.0 for components 1 and 2 (Fig. 3 and Table 2). These 26 metabolites were cross-referenced with the random forest data, which showed similar results. The PLSDA and random forest models were cross-validated—the permutation *p*-value for PLSDA and the out-of-the-box value for the random forest were both < 0.05. Furthermore, the random forest results showed that the TBM cases had 0% misclassification (no false negatives for the TBM group), while the control cases showed that 12% (3 out of the 24) were misclassified as TBM (see Table S1). For the univariate statistical measures, 50 metabolites had a Cohen’s effect size d-value > 0.5, 16 metabolites had an FDR *p*-value < 0.05, and 17 metabolites had a fold change > 1.5 (Fig. 3 and Table 2). A volcano plot (Fig. 3B) illustrates the metabolites with a statistically significant *p*-value < 0.05 and a fold change threshold > 1.5. A Venn diagram was generated (Fig. 3A) to illustrate the uniqueness and relatedness of the quantitative statistical results, based on t-test *p*-value < 0.05, effect size > 0.5, and VIP > 1.0. Lastly, the final list of metabolites for the metabolic model was selected under the condition that they conformed to three cut-off criteria [FDR *p*-value < 0.05, d-value > 0.5, and PLSDA VIP > 1.0 (Table 2)], which yielded 16 metabolites that will be discussed. Box plots of the 16 significant metabolites are in Fig. S3.

**Table 2:** Quantitative data table of important metabolites, based on effect size *d*-value > 0.5, t-test *p*-value < 0.05, or PLSDA VIP (Comp 1 and Comp 2) > 1.0. Also given in the table are the mean concentrations (micromole per liter; µmol/L), standard deviations (Stdev), and the metabolites. Control = Non-Neuro Non-Meningitis

Metabolites	Control Conc. (mean ± Stdev) in µmol/L	TBM Conc. (mean ± Stdev) in µmol/L	FDR *p*-value	Effects size *d*-value	PLSDA VIP value	
					Comp 1	Comp 2
*1-Butene, 1-butoxy-	0.00021 ± 0.0002	0	< 0.001	1.036	2.215	2.048
1-Deoxypentitol	0.0000044 ± 0.000062	0.0000015 ± 0.0000035	0.069	0.860	0.731	1.095
*1-Pentene	0.00019 ± 0.0002	0	< 0.001	1.103	2.057	1.897
*2-(2-Methoxyethoxy) acetic acid	0	0.00012 ± 0.00015	< 0.001	0.852	1.495	1.813
2,3,4-Trihydroxybutanoic acid	0.00198 ± 0.0045	0.00985 ± 0.0061	0.005	1.063	0.393	0.425
2-Desoxy-pentos-3-ulose	0.0000032 ± 0.0000073	0.00046 ± 0.00055	0.215	0.714	0.414	0.670
*2-Ethylhexanol	0.00042 ± 0.0007	0	< 0.001	0.643	1.829	1.693
2-Hydroxybutyric acid	0.00526 ± 0.0024	0.01204 ± 0.00766	0.902	0.782	0.017	0.086
*2-Ketoisocaproic acid	0	0.0000077 ± 0.0001	0.005	0.511	0.903	1.021
2-methoxyethyl acetate	0.000000974 ± 0.0000033	0.000466 ± 0.00097	0.002	0.468	1.235	1.333
*3,5,5-trimethylhexan-1-ol	0.0015 ± 0.0008	0	< 0.001	1.789	2.623	2.419
3-Hydroxybutyric acid	0.0418 ± 0.066	0.00587 ± 0.0108	0.008	0.721	0.375	0.414
*3-Oxaoct-4-en-2-imine	0.062 ± 0.033	0	< 0.001	1.867	2.869	2.645
5-Aminoimidazole	0.00194 ± 0.001203	0.00365 ± 0.00252	0.143	0.875	0.214	0.200
*5-Ethyl-1,2,4-oxadiazol-3-amine	0.00113 ± 0.0013	0	< 0.001	0.899	1.925	1.829
Arabinofuranose	0.0000043 ± 0.00011	0.00056 ± 0.00063	0.115	0.689	0.529	0.717
Citric acid	0.000202 ± 0.0000083	0.00038 ± 0.00027	0.232	0.517	0.152	0.167
Diethylene glycol	0.000203 ± 0.000132	0.00025 ± 0.00017	0.063	1.307	0.178	0.1652
*Erythrono-1, 4-lactone	0.00021 ± 0.0003	0	< 0.001	0.612	1.859	1.737
Ethanamine	0.0023 ± 0.0029	0.000008 ± 0.00013	< 0.001	0.799	0.589	0.545
Fructose	0.0031 ± 0.0016	0.00088 ± 0.00065	< 0.001	1.483	0.554	0.803
*Galactose	0.0000063 ± 0.0000059	0	< 0.001	1.068	1.187	1.129
Gluconic acid	0.0000423 ± 0.000446	0.00078 ± 0.00071	0.001	0.839	0.394	0.472
Glucose	0.0456 ± 0.0121	0.0216 ± 0.0178	< 0.001	1.625	0.528	0.694
Glyceric acid	0.0010 ± 0.0025	0.0062 ± 0.0021	< 0.001	1.973	0.698	0.6437
Glycine	0.00031 ± 0.0002	0.0067 ± 0.0083	< 0.001	0.797	0.716	0.8121
Glycolic acid	0.0048 ± 0.00023	0.00223 ± 0.00093	0.001	1.182	0.422	0.440
Hydroxypyruvic acid	0.0005 ± 0.00067	0.00152 ± 0.00054	0.805	1.177	0.034	0.095
L-(+)-Erythrulose	0.0014 ± 0.00075	0.00133 ± 0.00063	0.998	0.692	< 0.001	0.191
Lactic Acid	0.2464 ± 0.0788	0.4295 ± 0.3039	0.131	0.818	0.223	0.223
L-Aspartic acid	0.000471 ± 0.000799	0.00474 ± 0.00387	0.002	0.982	0.432	0.437
L-Proline	0	0.00156 ± 0.00262	0.262	0.591	0.628	0.659
L-Valine	0.000868 ± 0.000619	0.00925 ± 0.0158	0.004	0.538	0.414	0.493
Mannose	0.1116 ± 0.0326	0.0671 ± 0.0422	0.038	1.069	0.311	0.405
meso-Erythritol	0.01044 ± 0.00385	0.00847 ± 0.00449	0.016	0.791	0.269	0.422
*Methyl galactoside	0.00026 ± 0.0002	0	< 0.001	1.367	2.388	2.216
Octanoic acid	0.00167 ± 0.0012	0.000404 ± 0.000165	0.471	1.031	0.123	0.881
*Palmitic acid	0.000009 ± 0.000008	0	< 0.001	1.046	1.824	1.747
*Protein serine	0.0007 ± 0.0008	0	< 0.001	0.851	2.453	2.263
Pyroglutamic acid	0.0193 ± 0.0069	0.0412 ± 0.032	0.005	0.716	0.407	0.675
Ribitol	0.0249 ± 0.0097	0.01819 ± 0.00864	0.078	1.010	0.254	0.242
*Ribonolactone	0.00029 ± 0.00311	0	< 0.001	0.934	2.321	2.156
Ribose	0.00198 ± 0.00087	0.00178 ± 0.000919	0.038	0.689	0.242	0.289
Scyllo-Inositol	0.00162 ± 0.001042	0.00299 ± 0.00213	0.209	0.577	0.184	0.545
Surcose	0.0000013 ± 0.0000046	0.00014 ± 0.00137	0.009	1.005	0.852	0.799
*Tartronic acid	0.0021 ± 0.0012	0	< 0.001	1.381	2.558	2.359
tert-Butyldimethylsilyl 3-(acetylthio)propanoate	0.00000986 ± 0.000108	0.000341 ± 0.00039	0.676	0.605	0.129	0.631
*Undecylenic acid	0.00046 ± 0.0003	0	< 0.001	1.828	2.666	2.458

*Significant metabolite, where the criteria of *p*-value < 0.05, *d*-value > 0.5 and VIP > 1.0 were met

### Probable TBM and neuro non-meningitis groups—testing model robustness

**Fig. 4:**
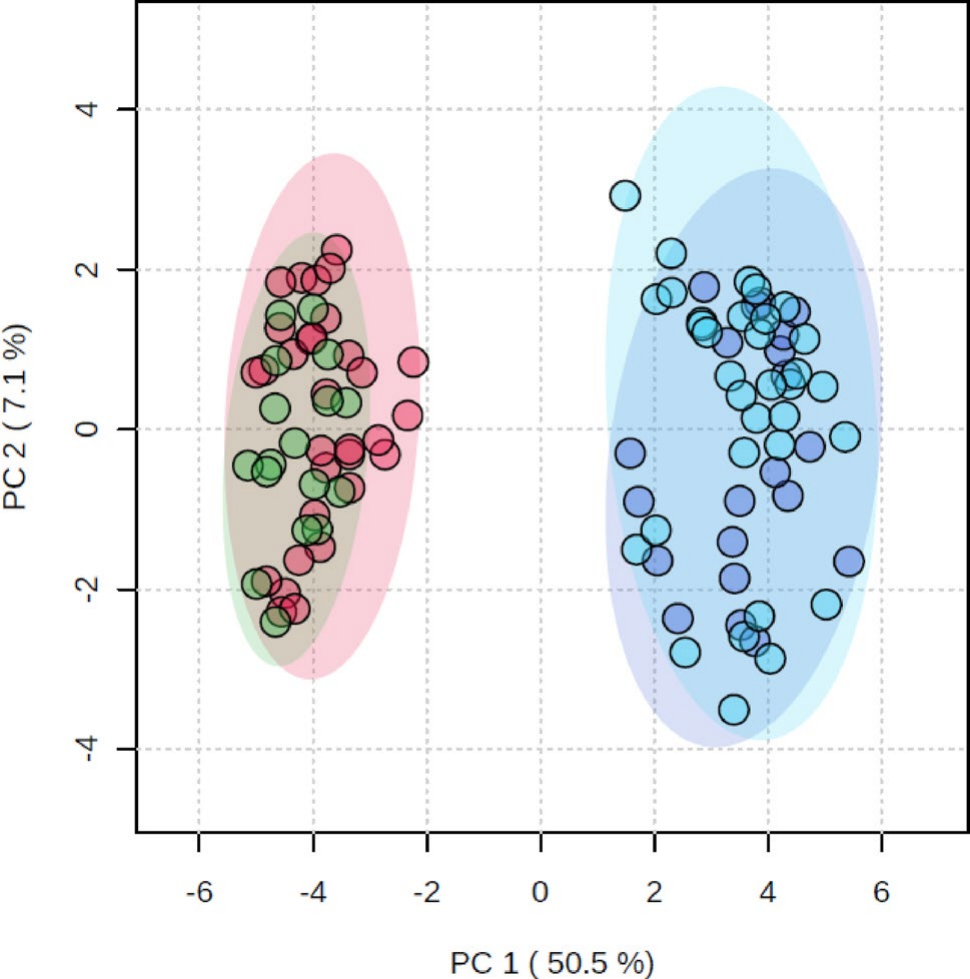
PCA scores plot of PCA 1 versus PCA 2 of the control groups (Non-Neuro Non-Meningitis [green] and Non-Neuro Non-Meningitis [red]) and the TBM groups (Def TBM [dark blue] and Prob TBM [light blue]). Only the 16 characterizing metabolites of our metabolic model of TBM were evaluated in this PCA. Natural separation between the control and TBM groups is visible. No samples lie outside the 95% confidence interval ellipses, and the overall explained variation is 57.6%

Two additional groups (Prob TBM and Neuro Non-Meningitis groups) were also investigated to evaluate the robustness of our metabolic model. The 16 metabolites identified as characterizing TBM, from the results on the differentiation of Def TBM against Non-Neuro Non-Meningitis, were used here. The PCA (Fig. 4) shows the overlap of all four groups and a total explained variation of 57.6%. Figure 4 shows the clear separation between the control groups (Non-Neuro Non-Meningitis and Neuro Non-Meningitis) and the TBM groups (Def TBM and Prob TBM). Hence, control cases with neurological symptoms cluster with the other controls without neurological symptoms. Similarly, the TBM groups overlap. Therefore, our metabolic model correctly classifies TBM and excludes all non-meningitis cases (Table 3).

**Table 3:** Quantitative data table of important metabolites as identified in Table 2, of the 2 control groups (Neuro Non-Meningitis and Non-Neuro Non-Meningitis) and the 2 experimental groups (Def TBM and Prob TBM). Also given in the table are the mean concentrations (micromole per liter; µmol/L) and standard deviations (Stdev) of the metabolites

	Neuro non-meningitis conc. (mean ± Stdev) in µmol/L	Non-neuro non-meningitis conc. (mean ± Stdev) in µmol/L	Def TBM conc. (mean ± Stdev) in µmol/L	Prob TBM conc. (mean ± Stdev) in µmol/L
1-Butene, 1-butoxy-	0.00035 ± 0.00021	0.00027 ± 0.00022	0 ± 0	0 ± 0
1-Deoxypentitol	0.00004 ± 0.00006	0.00004 ± 0.00007	0.00002 ± 0.00004	0.00001 ± 0.00002
1-Pentene	0.00025 ± 0.00036	0.00023 ± 0.00017	0 ± 0	0 ± 0
2-(2-Methoxyethoxy)acetic acid	0 ± 0	0 ± 0	0.00013 ± 0.00015	0.00009 ± 0.00012
2,3,4-Trihydroxybutanoic acid	0 ± 0	0 ± 0	0.00986 ± 0.006	0.00878 ± 0.00588
2-Desoxy-pentos-3-ulose	0 ± 0	0 ± 0	0.00046 ± 0.00054	0.00052 ± 0.00059
2-Ethylhexanol	0.00026 ± 0.0005	0.00036 ± 0.00053	0 ± 0	0 ± 0
2-Hydroxybutyric acid	0.005 ± 0.0032	0.00513 ± 0.0026	0.01205 ± 0.00748	0.00929 ± 0.00501
2-Ketoisocaproic acid	0 ± 0	0 ± 0	0.00008 ± 0.0001	0.00004 ± 0.00007
2-methoxyethyl acetate	0.00004 ± 0.0001	0.00003 ± 0.0001	0 ± 0	0 ± 0
3,5,5-trimethylhexan-1-ol	0.0019 ± 0.00051	0.00174 ± 0.00051	0 ± 0	0 ± 0
3-Hydroxybutyric acid	0.01839 ± 0.02756	0.05517 ± 0.07554	0.00587 ± 0.01053	0.00578 ± 0.01609
3-Oxaoct-4-en-2-imine	0.07104 ± 0.02147	0.07376 ± 0.02007	0 ± 0	0 ± 0
5-Aminoimidazole	0 ± 0	0 ± 0	0.00057 ± 0.00071	0.00048 ± 0.00064
5-Ethyl-1,2,4-oxadiazol-3-amine	0.00085 ± 0.00117	0.0015 ± 0.0013	0 ± 0	0 ± 0
Arabinofuranose	0 ± 0	0 ± 0	0.00056 ± 0.00061	0.00028 ± 0.00027
Citric acid	0.00022 ± 0.00008	0.00018 ± 0.00005	0.00038 ± 0.00027	0.00034 ± 0.00016
Diethylene glycol	0.00014 ± 0.00011	0.00019 ± 0.00012	0.00025 ± 0.00017	0.00027 ± 0.00017
Erythrono-1,4-lactone	0.00019 ± 0.00041	0.0003 ± 0.0004	0 ± 0	0 ± 0
Ethanamine	0.00192 ± 0.00288	0.00256 ± 0.00289	0.00009 ± 0.00013	0.00009 ± 0.00021
Fructose	0.00303 ± 0.00146	0.00266 ± 0.00129	0.00088 ± 0.00064	0.0012 ± 0.00105
Galactose	0.00007 ± 0.00009	0.00008 ± 0.00006	0 ± 0	0 ± 0
Gluconic acid	0.00073 ± 0.00064	0.00035 ± 0.00031	0.00079 ± 0.00069	0.00109 ± 0.00066
Glucose	0.04221 ± 0.01313	0.04258 ± 0.01061	0.02165 ± 0.0174	0.02681 ± 0.01746
Glyceric acid	0 ± 0	0 ± 0	0.00625 ± 0.00208	0.00699 ± 0.00338
Glycine 3	0.00026 ± 0.00016	0.0003 ± 0.00025	0.00674 ± 0.00809	0.00502 ± 0.00584
Glycolic acid	0.00561 ± 0.00196	0.0055 ± 0.00223	0.00229 ± 0.0009	0.00205 ± 0.00094
Hydroxypyruvic acid	0.0005 ± 0.00062	0.0005 ± 0.00068	0.00153 ± 0.00053	0.00152 ± 0.0008
L-( +)-Erythrulose	0.00115 ± 0.00072	0.00157 ± 0.0007	0.00133 ± 0.00061	0.00114 ± 0.00067
Lactic acid	0.20264 ± 0.10693	0.24801 ± 0.06653	0.42959 ± 0.29659	0.4371 ± 0.27626
L-Aspartic acid	0.00043 ± 0.00083	0.00044 ± 0.00088	0.00474 ± 0.00378	0.00421 ± 0.00424
L-Proline	0 ± 0	0 ± 0	0.00157 ± 0.00256	0.00153 ± 0.00229
L-Valine 2 TMS	0.00055 ± 0.00042	0.00086 ± 0.00064	0.00925 ± 0.01542	0.00599 ± 0.00779
Mannose	0.10239 ± 0.02074	0.09833 ± 0.01854	0.06712 ± 0.04119	0.09233 ± 0.04479
meso-Erythritol	0.01159 ± 0.01144	0.0104 ± 0.00426	0.00847 ± 0.00438	0.00957 ± 0.00999
Methyl galactoside	0.00031 ± 0.00019	0.00032 ± 0.00018	0 ± 0	0 ± 0
Octanoic acid	0.00215 ± 0.00096	0.00206 ± 0.00117	0.0004 ± 0.00016	0.00041 ± 0.0002
Palmitic acid	0.00006 ± 0.00008	0.00009 ± 0.00008	0 ± 0	0 ± 0
Protein serine	0.00121 ± 0.00089	0.00084 ± 0.00089	0 ± 0	0 ± 0
Pyroglutamic acid	0.01716 ± 0.00842	0.01869 ± 0.00769	0.04129 ± 0.03166	0.0436 ± 0.0299
Ribitol	0.02329 ± 0.01106	0.02407 ± 0.0101	0.0182 ± 0.00844	0.02153 ± 0.01063
Ribonolactone	0.00025 ± 0.00039	0.00034 ± 0.00032	0 ± 0	0 ± 0
Ribose	0.00188 ± 0.00108	0.00212 ± 0.00092	0.00178 ± 0.0009	0.00183 ± 0.00118
Scyllo-Inositol	0.00268 ± 0.00278	0.00175 ± 0.00121	0.003 ± 0.00208	0.00245 ± 0.00182
Sucrose	0 ± 0	0 ± 0	0.00148 ± 0.00134	0.00324 ± 0.00502
Tartronic acid	0.00038 ± 0.00044	0.00053 ± 0.00026	0 ± 0	0 ± 0
tert-Butyldimethylsilyl-3-(acetylthio)propanoate	0.00028 ± 0.00025	0.0001 ± 0.00012	0.00034 ± 0.00038	0.00041 ± 0.00039
Undecylenic acid	0.00237 ± 0.00064	0.00238 ± 0.00063	0 ± 0	0 ± 0

## Discussion

**Fig. 5:**
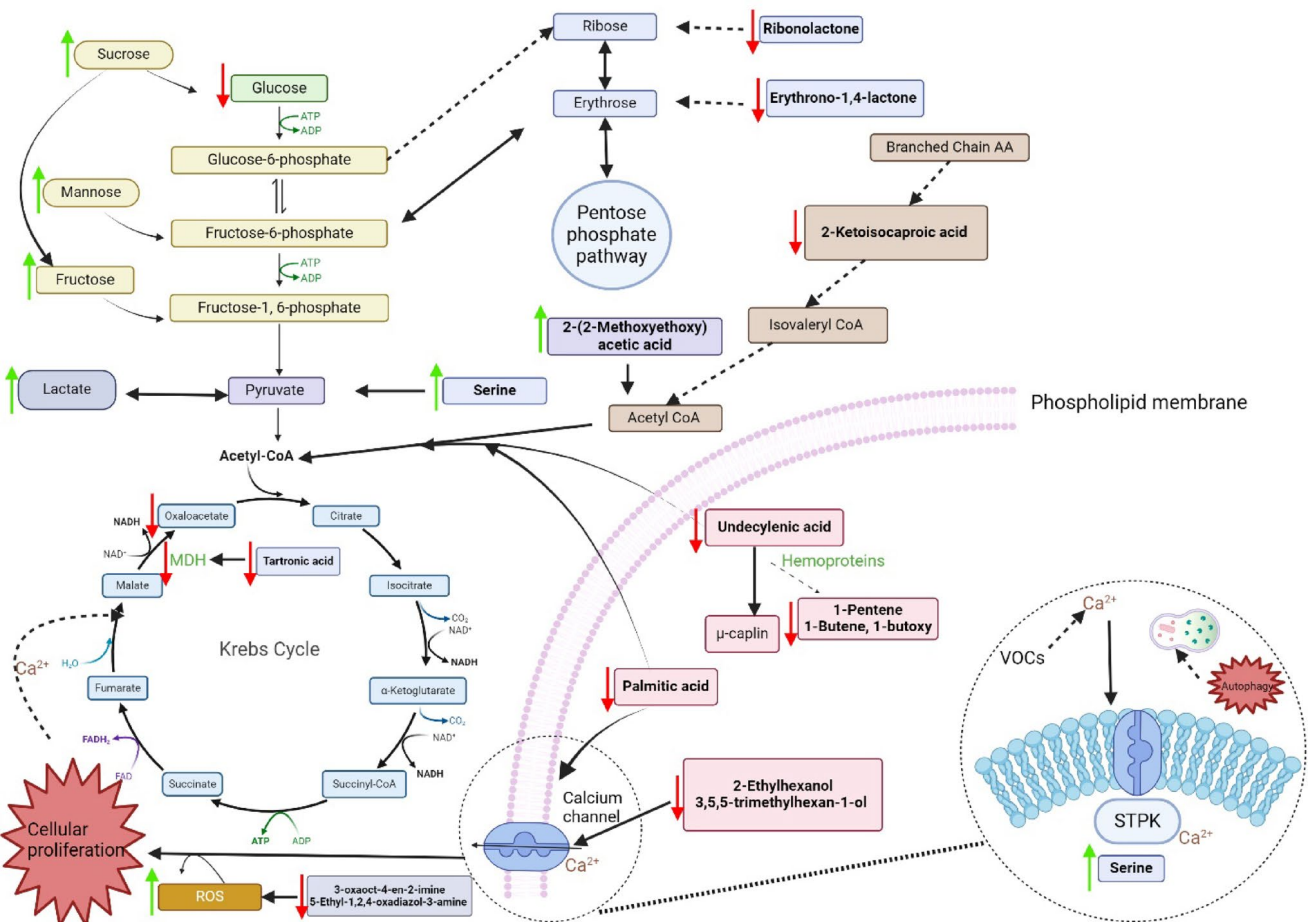
Schematic of a metabolic model describing *M.tb* infection in the brain. Pathways affected are color-coded, where light blue indicates the Krebs cycle, pink indicates fatty acid metabolism and volatile organic compounds (VOCs), brown shows the branched-chain amino acids, purple represents the pentose phosphate pathway, and yellow indicates glycolysis. The red arrows represent downregulation of metabolites by TBM, and the green arrows show upregulation. List of abbreviations: AA, amino acids; ADP, adenosine diphosphate; ATP, adenosine triphosphate; STPK, serine/threonine protein kinases; ROS, reactive oxygen species

For this study, CSF from 24 control and 21 Def TBM cases were analyzed via GCxGC-TOFMS and underwent statistical analyses. For a metabolite to be considered significant (for characterization purposes), it had to conform to the following cutoff criteria: *p*-value < 0.05, *d*-value > 0.5, and VIP > 1.0. A total of 16 significant metabolites were identified in this study (Table 2). In addition to these 16 significant metabolites, 32 metabolites had a *d*-value > 0.5 and a VIP > 1.0 but did not have a *p*-value < 0.05 and are also described in Table 2. Metabolites discussed are found in glucose metabolism (galactose; erythrono-1,4-lactone; ribonolactone; tartronic acid), amino acid metabolism (protein serine and 2-ketoisocaproic acid), fatty acid metabolism (undecylenic acid and palmitic acid), imines (3-oxaoct-4-en-2-imine), volatile organic compounds (2-ethylhexanol and 3,5,5-trimethylhexan-1-ol), and various alkenes (1-butene, 1-butoxy-1 butene; 1-pentene;2-(2-methoxyethoxy) acetic acid), amines (5-ethyl-1,2,4-oxadiazol-3-amine) and glycosides (methyl galactose). The metabolic roles of these metabolites in TBM are described below and illustrated as a metabolic model in Fig. 5.

### Metabolic burst

The presence of elevated levels of lactate (lactic acid), sucrose, and 2-(2-methoxyethoxy) acetic acid, accompanied by reduced levels of glucose and galactose, is all indicative of a metabolic burst—an increase in energy production in the *M.tb*-infected brain, previously described in TBM [7, 12–14]. The significantly reduced levels of glucose, accompanied by increased levels of lactic acid, also support the hypothesis of the astrocyte-microglia lactate shuttle (AMLS) [15]. Briefly, glucose levels in the brain decrease during *M.tb* infection because of increased glycolysis in astrocytes. The glycolytic end-product pyruvate is converted to lactic acid, which is then shuttled from astrocytes to the microglia—the resident immune cell of the brain. Hence, brain energy metabolism becomes focused on fighting the *M.tb* infection and protecting neurons.

Reduced concentrations of tartronic acid, ribonolactone, and erythron-1,4-lactone in the CSF also support the concept of metabolic burst in TBM patients. Ribonolactone (also known as D-ribono-1,4-lactone) is the oxidized form of ribose and erythrono-1,4-lactone (also known as D-erythronolactone), which is derived from erythose. Ribose and erythrose are both involved in various biological processes including the pentose phosphate pathway and glucose metabolism, where they are used in nucleotide and amino acid synthesis [16]. During glucose metabolism, ribose-5-phosphate and erythrose-4-phosphate can be converted to glucose-6-phosphate, which is then catabolized via the glycolytic pathway, thereby contributing to the metabolic burst. Tartronic acid is a lactate dehydrogenase inhibitor known to block glycolysis, and it also inhibits cytosolic malate dehydrogenase [17, 18]. Thus, reduced tartronic acid in the *M.tb*-infected brain can result in an increased metabolic burst.

Lastly, significantly elevated levels of 2-ketoisocaproic acid were detected in the CSF of the TBM patient group. 2-Ketoisocaproic acid is formed during the catabolism of leucine (a branched-chain amino acid), serves as an intermediate for the synthesis of glutamate, and can also be utilized in the Krebs cycle for energy production. Hence, significantly increased 2-ketoisocaproic acid further supports the metabolic burst in TBM.

### Calcium signaling

This study found two volatile organic compounds (VOCs)—2-ethylhexanol and 3,5,5-trimethylhexan-1-ol, significantly reduced to below detectable concentrations in the TBM group, compared to that in the Non-Neuro Non-Meningitis group. Studies showed that 3,5,5-trimethylhexan-1-ol has been found to induce calcium signaling and cAMP in cell lines, where intracellular calcium is dependent on the cAMP-dependent signaling pathway [19]. *M.tb* has been shown to inhibit calcium signaling within the body, and it’s this inhibition that results in susceptibility to *M.tb* infection, as well as *M.tb* survival and growth [20]. Intracellular calcium also plays a role in autophagy by activating the serine/threonine kinase enzyme, which in turn leads to the phosphorylation of beclin-1 and VPS34, inducing autophagy [21]. Autophagy serves to contain *M.tb* infection by preventing the fusion of the phagosomes with the lysosomes [22]. Considering this, the inhibition of calcium signaling by *M.tb* in turn results in the inhibition of these VOCs. Additionally, protein serine was found to have significantly reduced concentrations (non-detectable) in the TBM group compared to that of the Non-Neuro Non-Meningitis cases (0.0007 ± 0.0008 µmol/L). Protein serine concentrations in TBM are indicative of the serine/threonine protein kinases (STPKs) found in *M.tb*, which act as part of the mechanism for signal transduction, allowing for growth/survival in the host [23]. Thus, the significantly decreased concentrations of 2-ethylhexanol, 3,5,5-trimethylhexan-1-ol, and protein serine in the TBM group support the hypothesis of *M.tb*-inhibited calcium signaling; however, this is completely speculative at this point and requires future validation studies.

Moreover, it should be noted that VOCs, such as alkanes, are a natural component of the myelin sheath in neurons [24]. Lipid peroxidation, common in TBM, induces alterations in the physical properties of myelin membranes, releasing VOCs [25]. In a study on multiple sclerosis (MS) [26], a disease known for degradation of the myelin sheaths, exhaled VOCs were identified as potential markers of MS (i.e., degradation of the myelin sheath releases VOCs). In fact, some Mycobacterium species have been shown to directly consume alkanes as a source of carbon [27–29]. Hence, we postulate that the complete absence of some VOCs in the CSF of TBM cases could be due to the demyelination of neurons, contributing to neuronal damage that is typically associated with TBM.

### Fatty acid and alkene metabolism

Significantly reduced concentrations to below the levels of detection for undecylenic acid (UDA) and palmitic acid were identified as key metabolites in this study in the TBM group compared to the Non-Neuro Non-Meningitis group. UDA has been shown to have a neuroprotective effect against neural apoptosis induced by glutamate, hydrogen peroxide, and amyloid beta protein [30]. UDA forms part of the phospholipid layers found in cell membranes of the brain and acts by reducing µ-calpain activity (a type of calcium-activated cysteine protease enzymes where µ-calpain are activated by an influx of calcium ions) [30–32]. Caplins are known to play a role in apoptosis by cleaving and activating pro-apoptotic proteins, such as p35, which has an adverse impact on neuron structure and function, potentially leading to neurological decline [30]. Hence, it is likely that the lack of UDA in the TBM group could be an indication of increased neurological deficits associated with TBM due to the decreased caplin activity brought on by the decreased UDA levels. Palmitic acid contributes 20–30% of the total fatty acids found in membrane phospholipids and plays a crucial role in membrane function by assisting with cell division, intracellular membrane movement, signal transduction, and influencing the physical properties of the membrane. Changes in fatty acids are also shown to alter the fluidity of membranes, which has previously been associated with several immune disorders and neurological diseases [33]. Previous research also shows that *M.tb* utilizes host-derived lipids, including palmitic acid, for replication using beta-oxidation [34].

Unique to our metabolomics investigation, we are reporting altered alkene markers in the CSF of TBM patients. Grant et al*.* [35] showed that alkenes, such as 1-pentene and 1-butene, are formed during fatty acid metabolism via various enzymatic reactions. The enzymes involved in this process use hemoproteins, including cytochrome P450, which are typically involved in oxygenations. Cytochromes (CYP1B1 and CYP2B) are expressed in the blood–brain barrier (BBB), where they have been shown to have neuroprotective effects on the brain by working in conjunction with the efflux transports in the brain [35, 36]. Other research has also shown that in neurons, cytochromes are involved in neurotransmitter synthesis [37].

The significantly reduced concentrations of fatty acids (used as a substrate for *M.tb* growth and replication) and alkenes in the TBM group, and the subsequent alteration of the cellular membranes in the brain, are likely a further contributor to the neurological symptoms seen in TBM patients.

### Imine, amines, and methyl galactoside

In this study, 3-oxaoct-4-en-2-imine and 5-ethyl-1.2.4-oxadiazol-3-amine also occurred in significantly reduced concentrations in the TBM group compared to the Non-Neuro Non-Meningitis group. Limited research exists describing these compounds; however, quinone imines have been previously associated with cytotoxicity, redox reactions, and ROS formation in cancer in various in vitro studies [38, 39]. Biernacki et al. [40] described 1,2,4-oxadiazoles as having anticancer, antimicrobial, and anti-inflammatory functions. Other research has highlighted that the derivatives of 1,2,4-oxadiazoles exhibit antitubercular action, specifically against *M.tb* [41, 42]. Preclinical research in fecal metabolomics has also shown that imines are upregulated in mice after oral ingestion of the probiotic *Lacticaseibacillus rhamnosus* IDCC 3201 and that this probiotic also has neuroprotective properties [43]. Hence, imines in the CSF may also serve in a neuroprotective capacity and/or function in energy production through redox reactions. The reduced levels of these amines and imines in the CSF of TBM patients are likely due to their inhibition/depletion due to the *M.tb* infection and subsequently contribute to the neurological symptoms seen in these patients.

Methyl galactoside concentrations were significantly reduced in the CSF of the TBM group. Current research indicates that glycosides have a neuroprotective effect, where they can modulate neuroinflammation by targeting the BBB to reduce neuroinflammation and by decreasing the expression of inflammatory markers in glial cells [44, 45].Thus, the depletion of methyl galactoside in the TBM group may be brought on by *M.tb*, thereby contributing to the neurological complications associated with TBM. Additionally, this could be a potential therapeutic target for the management of TBM.

### Testing the robustness of the metabolic markers and their possible diagnostic use

Figure 5 is a schematic that explains the 16 significant metabolites and some of the other identified metabolic of this study, in terms of metabolic pathways. Figure 5 is thus a metabolic model describing *M.tb* infection in the brain from this study. To test the robustness of this metabolic model, we analyzed the CSF derived from participants diagnosed as Prob TBM and the Neuro control group. Based on the results (PCA—Fig. 4), the two control groups overlap with each other, the two TBM groups overlap with each other, and there is a distinct separation between the control and TBM groups. This means that our metabolic model, which describes both probable and definite TBM similarly, is unique from clinical neurological symptoms (such as seizures—febrile, epilepsy, and viral encephalopathy). We recommend further investigating the significant metabolites detected in this study in the CSF of TBM patients for diagnostic applications.

Further inspection of the 9 metabolites that are only present in the TBM groups (Table S2) shows that three metabolites (2,3,4-trihydroxybutanoic acid; 2-desoxy-pentos-3-ulose and glyceric acid) were present in all samples across all stages of TBM. Of note, 2-ketoisocaproic acid and L-proline were significantly lower (if not absent) in the stage 1 TBM samples, arabinofuranose levels were lower in stage 2a samples only, 5-aminoimidazole was low in samples with stage 2b, and for stage 3 samples, 2-(2-methoxyethoxy)acetic acid levels were completely depleted. This suggests further application of these metabolite markers for not only aiding in diagnosing TBM but also for determining the stages of TBM. However, since the number of cases per TBM stage was too small in this study to draw conclusive results, we recommend doing a larger-scale study with more cases (> 20) per TBM stage to investigate the role of metabolites in differentiating TBM stage.

## Conclusion

This is the first study to use a GCxGC-TOFMS metabolomics research approach to characterize TBM and yielded 48 metabolites of interest. There are 6 metabolites (2-hydroxybutyric acid, glucose, valine, lactate (lactic acid), citrate (citric acid), and proline) identified in this study that were also previously reported in a ^1^H-NMR study on the same cohort, highlighting the complementary nature between ^1^H-NMR and GCxGC-TOFMS. Eight new metabolites that have not previously been associated with TBM are newly reported in this study. This study supports previous research and confirms the AMLS hypothesis and metabolic burst previously described as occurring in TBM. Some of the affected metabolic pathways described in this study are glycolysis, pentose phosphate pathway, amino acids, and fatty acid metabolism. New information is provided on the effect of TBM on neurometabolism and the resulting neurological symptoms typically associated with *M.tb* infection in the brain. The robustness of the proposed metabolic model was shown by testing controls presenting neurological symptoms and experimental samples that were inconclusive for TBM. Further research into the diagnostic capacity of the 16 metabolites characterizing TBM in this study should be considered a future objective.

## Supplementary Information

Below is the link to the electronic supplementary material.

Supplementary file1 (DOCX 445 kb)

## Data Availability

Data will be made available on request.

## References

[1] Thwaites G, Chau T, Mai N, Drobniewski F, McAdam K, Farrar J (2000) Tuberculous meningitis. J Neurol Neurosurg Psychiatry 68(3):289–299 10675209 10.1136/jnnp.68.3.289 PMC1736815

[2] Garg R (2010) Tuberculous meningitis. Acta Neurol Scand 122(2):75–90 20055767 10.1111/j.1600-0404.2009.01316.x

[3] Wishart DS, Lewis MJ, Morrissey JA, Flegel MD, Jeroncic K, Xiong Y et al (2008) The human cerebrospinal fluid metabolome. J Chromatogr B Analyt Technol Biomed Life Sci 871(2):164–173 18502700 10.1016/j.jchromb.2008.05.001

[4] Marx GE, Chan ED (2011) Tuberculous meningitis: diagnosis and treatment overview. Tuberculosis Res Treat 2011(1):798764 10.1155/2011/798764 PMC3335590 22567269

[5] Kent SJ, Crowe SM, Yung A, Lucas CR, Mijch AM (1993) Tuberculous meningitis: a 30-year review. Clin Infect Dis 17(6):987–994 8110957 10.1093/clinids/17.6.987

[6] Mason S, van Furth AM, Mienie LJ, Engelke UF, Wevers RA, Solomons R et al (2015) A hypothetical astrocyte–microglia lactate shuttle derived from a 1 H NMR metabolomics analysis of cerebrospinal fluid from a cohort of South African children with tuberculous meningitis. Metabolomics 11:822–837 26109926 10.1007/s11306-014-0741-z PMC4475545

[7] Van Zyl CDW, Solomons R, Van Reenen M, Mason S (2020) Metabolic characterization of tuberculous meningitis in a South African paediatric population using 1H NMR metabolomics. J Infect 81(5):743–752 32712206 10.1016/j.jinf.2020.06.078

[8] Zhang P, Zhang W, Lang Y, Qu Y, Chu F, Chen J et al (2018) Mass spectrometry-based metabolomics for tuberculosis meningitis. Clin Chim Acta 483:57–63 29678632 10.1016/j.cca.2018.04.022

[9] Marais S, Thwaites G, Schoeman JF, Török ME, Misra UK, Prasad K, Donald PR, Wilkinson RJ, Marais BJ (2010) Tuberculous meningitis: a uniform case definition for use in clinical research. The Lancet infectious diseases 10(11):803–812 10.1016/S1473-3099(10)70138-9 20822958

[10] Luier L, Loots DT (2016) Tuberculosis metabolomics reveals adaptations of man and microbe in order to outcompete and survive. Metabolomics 12:1–9

[11] Wang S-Y, Kuo C-H, Tseng YJ (2013) Batch normalizer: a fast total abundance regression calibration method to simultaneously adjust batch and injection order effects in liquid chromatography/time-of-flight mass spectrometry-based metabolomics data and comparison with current calibration methods. Anal Chem 85(2):1037–1046 23240878 10.1021/ac302877x

[12] Mason S, van Furth AM, Mienie LJ, Engelke UF, Wevers RA, Solomons R et al (2015) A hypothetical astrocyte-microglia lactate shuttle derived from a (1)H NMR metabolomics analysis of cerebrospinal fluid from a cohort of South African children with tuberculous meningitis. Metabolomics 11(4):822–837 26109926 10.1007/s11306-014-0741-z PMC4475545

[13] Mason S, Solomons R (2021) CSF metabolomics of tuberculous meningitis. Metabolites 11(10):661 34677376 10.3390/metabo11100661 PMC8541251

[14] Isaiah S, Loots DT, van Reenen M, Solomons R, van Elsland S, Tutu van Furth AM et al (2024) Urinary metabolic characterization of advanced tuberculous meningitis cases in a South African paediatric population. Front Mol Biosci 11:1253983 38560518 10.3389/fmolb.2024.1253983 PMC10978807

[15] Mason S (2017) Lactate shuttles in neuroenergetics-homeostasis, allostasis and beyond. Front Neurosci 11:43 28210209 10.3389/fnins.2017.00043 PMC5288365

[16] Stincone A, Prigione A, Cramer T, Wamelink MM, Campbell K, Cheung E et al (2015) The return of metabolism: biochemistry and physiology of the pentose phosphate pathway. Biol Rev Camb Philos Soc 90(3):927–963 25243985 10.1111/brv.12140 PMC4470864

[17] Fiume L, Manerba M, Vettraino M, Di Stefano G (2010) Impairment of aerobic glycolysis by inhibitors of lactic dehydrogenase hinders the growth of human hepatocellular carcinoma cell lines. Pharmacology 86(3):157–162 20699632 10.1159/000317519

[18] Fiume L (1960) Inhibition of aerobic glycolysis in Yoshida ascites hepatoma by tartronic acid. Nature 187(4739):792–793 13823333 10.1038/187792a0

[19] Tsai T, Veitinger S, Peek I, Busse D, Eckardt J, Vladimirova D et al (2017) Two olfactory receptors—OR2A4/7 and OR51B5—differentially affect epidermal proliferation and differentiation. Exp Dermatol 26(1):58–65 27315375 10.1111/exd.13132

[20] Liu F, Chen J, Wang P, Li H, Zhou Y, Liu H et al (2018) Microrna-27a controls the intracellular survival of Mycobacterium tuberculosis by regulating calcium-associated autophagy. Nat Commun 9(1):4295 30327467 10.1038/s41467-018-06836-4 PMC6191460

[21] Wong P-M, Puente C, Ganley IG, Jiang X (2013) The ULK1 complex: sensing nutrient signals for autophagy activation. Autophagy 9(2):124–137 23295650 10.4161/auto.23323 PMC3552878

[22] Stanley SA, Cox JS (2013) Host–pathogen interactions during Mycobacterium tuberculosis infections. Pathog Mycobacterium Tuberc Interact Host Org 2013:211–241 10.1007/82_2013_332 23881288

[23] Prisic S, Husson RN (2014) Mycobacterium tuberculosis serine/threonine protein kinases. Mol Genet Mycobact 2014:681–708 10.1128/microbiolspec.MGM2-0006-2013 PMC4242435 25429354

[24] Bourre JM, Cassagne C, Larrouquere-Regnier S, Darriet D (1977) Occurrence of alkanes in brain myelin. Comparison between normal and quaking mouse. J Neurochem 29(4):645–648 591942 10.1111/j.1471-4159.1977.tb07781.x

[25] Surewicz WK, Epand RM, Epand RF, Hallett FR, Moscarello MA (1986) Modulation of myelin basic protein-induced aggregation and fusion of liposomes by cholesterol, aliphatic aldehydes and alkanes. Biochim Biophys Acta (BBA) 863(1):45–52 2430621 10.1016/0005-2736(86)90385-8

[26] Broza YY, Har-Shai L, Jeries R, Cancilla JC, Glass-Marmor L, Lejbkowicz I et al (2017) Exhaled breath markers for nonimaging and noninvasive measures for detection of multiple sclerosis. ACS Chem Neurosci 8(11):2402–2413 28768105 10.1021/acschemneuro.7b00181

[27] Dunlap K, Perry J (1968) Effect of substrate on the fatty acid composition of hydrocarbon-and ketone-utilizing microorganisms. J Bacteriol 96:318–321 16562157 10.1128/jb.96.2.318-321.1968 PMC252300

[28] Dunlap K, Perry J (1967) Effect of substrate on the fatty acid composition of hydrocarbon-utilizing microorganisms. J Bacteriol 94:1919–1923 6074400 10.1128/jb.94.6.1919-1923.1967 PMC276922

[29] Churchill SA, Harper JP, Churchill PF (1999) Isolation and characterization of a *Mycobacterium* species capable of degrading three-and four-ring aromatic and aliphatic hydrocarbons. Appl Enviro Microbiol 65:549–552 10.1128/aem.65.2.549-552.1999 PMC91060 9925581

[30] Lee E, Eom JE, Kim HL, Kang DH, Jun KY, Jung DS et al (2012) Neuroprotective effect of undecylenic acid extracted from *Ricinus**communis* L. through inhibition of mu-calpain. Eur J Pharm Sci 46(1–2):17–25 22333440 10.1016/j.ejps.2012.01.015

[31] Wei J-W, Yang L-M, Sun SH, Chiang C-L (1987) Phospholipids and fatty acid profile of brain synaptosomal membrane from normotensive and hypertensive rats. Int J Biochem 19(12):1225–1228 3436482 10.1016/0020-711x(87)90107-8

[32] Jantas D, Piotrowski M, Lason W (2015) An involvement of PI3-K/Akt activation and inhibition of AIF translocation in neuroprotective effects of undecylenic acid (UDA) against pro-apoptotic factors-induced cell death in human neuroblastoma SH-SY5Y cells. J Cell Biochem 116(12):2882–2895 26012840 10.1002/jcb.25236

[33] Carta G, Murru E, Banni S, Manca C (2017) Palmitic acid: physiological role, metabolism and nutritional implications. Front Physiol 8:902 29167646 10.3389/fphys.2017.00902 PMC5682332

[34] Cole S, Brosch R, Parkhill J, Garnier T, Churcher C, Harris D et al (1998) Deciphering the biology of Mycobacterium tuberculosis from the complete genome sequence. Nature 396(6707):190 10.1038/31159 9634230

[35] Grant JL, Hsieh CH, Makris TM (2015) Decarboxylation of fatty acids to terminal alkenes by cytochrome P450 compound I. J Am Chem Soc 137(15):4940–4943 25843451 10.1021/jacs.5b01965

[36] Miksys S, Rao Y, Hoffmann E, Mash DC, Tyndale RF (2002) Regional and cellular expression of CYP2D6 in human brain: higher levels in alcoholics. J Neurochem 82(6):1376–1387 12354285 10.1046/j.1471-4159.2002.01069.x

[37] Hiroi T, Imaoka S, Funae Y (1998) Dopamine formation from tyramine by CYP2D6. Biochem Biophys Res Commun 249(3):838–843 9731223 10.1006/bbrc.1998.9232

[38] Powis G, Hodnett EM, Santone KS, See KL, Melder DC (1987) Role of metabolism and oxidation-reduction cycling in the cytotoxicity of antitumor quinoneimines and quinonediimines. Cancer Res 47(9):2363–2370 3032421

[39] Klopcic I, Dolenc MS (2018) Chemicals and drugs forming reactive quinone and quinone imine metabolites. Chem Res Toxicol 32(1):1–34 30500181 10.1021/acs.chemrestox.8b00213

[40] Biernacki K, Daśko M, Ciupak O, Kubiński K, Rachon J, Demkowicz S (2020) Novel 1, 2, 4-oxadiazole derivatives in drug discovery. Pharmaceuticals 13(6):111 10.3390/ph13060111 PMC7345688 32485996

[41] Upare AA, Gadekar PK, Sivaramakrishnan H, Naik N, Khedkar VM, Sarkar D et al (2019) Design, synthesis and biological evaluation of (E)-5-styryl-1, 2, 4-oxadiazoles as anti-tubercular agents. Bioorg Chem 86:507–512 30776681 10.1016/j.bioorg.2019.01.054

[42] dos Santos Filho JM, Macedo TS, Teixeira HMP, Moreira DRM, Challal S, Wolfender J-L et al (2016) Conjugation of N-acylhydrazone and 1, 2, 4-oxadiazole leads to the identification of active antimalarial agents. Bioorg Med Chem 24(22):5693–5701 27667552 10.1016/j.bmc.2016.09.013

[43] Song JG, Mun D, Lee B, Song M, Oh S, Kim J-M et al (2023) Protective effects of Lacticaseibacillus rhamnosus IDCC3201 on motor functions and anxiety levels in a chronic stress mouse model. Food Sci Anim Resour 43(6):1044 37969325 10.5851/kosfa.2023.e54 PMC10636227

[44] Jansson D, Dieriks VB, Rustenhoven J, Smyth LC, Scotter E, Aalderink M et al (2021) Cardiac glycosides target barrier inflammation of the vasculature, meninges and choroid plexus. Commun Biol 4(1):260 33637884 10.1038/s42003-021-01787-x PMC7910294

[45] Yu L, Chen C, Wang L-F, Kuang X, Liu K, Zhang H et al (2013) Neuroprotective effect of kaempferol glycosides against brain injury and neuroinflammation by inhibiting the activation of NF-κB and STAT3 in transient focal stroke. PLoS ONE. 10.1371/journal.pone.0055839 23437066 10.1371/journal.pone.0055839 PMC3577792

