# Supplementary material for: Characterizing tuberculous meningitis in a South African pediatric cohort using GCxGC-TOFMS metabolomics
Source: Med Microbiol Immunol. 2025 Sep 27;214(1):46. doi: 10.1007/s00430-025-00857-9 (PMC12476309; doi:10.1007/s00430-025-00857-9)
Supplement: Supplementary file 1 — Supplementary file1 (DOCX 445 kb) [file 430_2025_857_MOESM1_ESM.docx]

**Supplementary data:**

## **Characterizing tuberculous meningitis in a South African paediatric cohort using GCxGC-TOFMS metabolomics**

**Anouska Mangaroo-Pillay¹, Du Toit Loots¹, Regan Solomons^2^, Shayne Mason¹***

¹ Human Metabolomics, Faculty of Natural and Agricultural Sciences, North-West University, Potchefstroom, South Africa.

^2^ Department of Paediatrics and Child Health, Faculty of Medicine and Health Sciences, Stellenbosch University, Cape Town, South Africa.


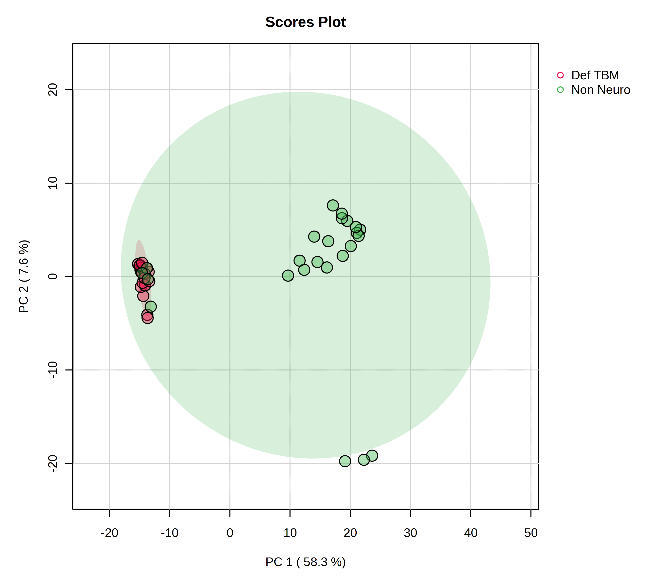


**Fig. S1** PCA scores plot of PCA 1 vs PCA 2 of the definite TBM [Def TBM (n=21); red] and control [Non-Neuro Non-Meningitis (n=25); green] groups.


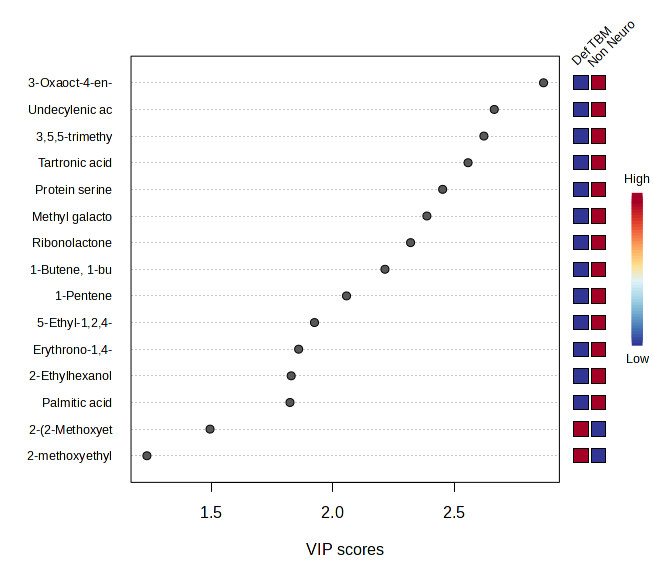


**Fig.S2** PLS-DA of the finalized data set, representing the metabolites found in the samples against the VIP scores

| 3-Oxaoct-4-en-2-imine  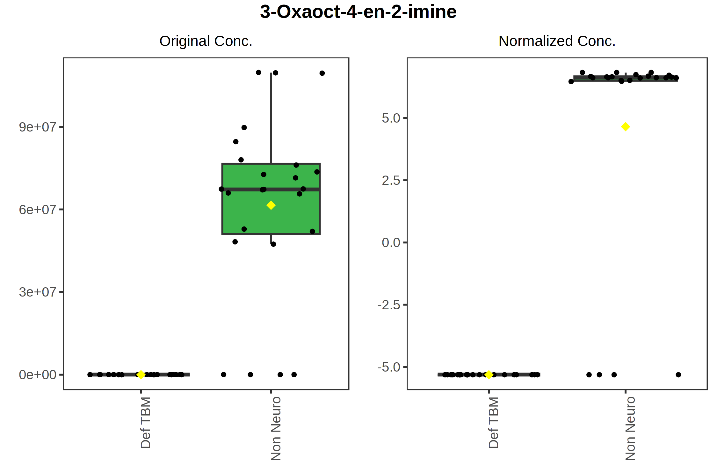 | Undecylenic acid  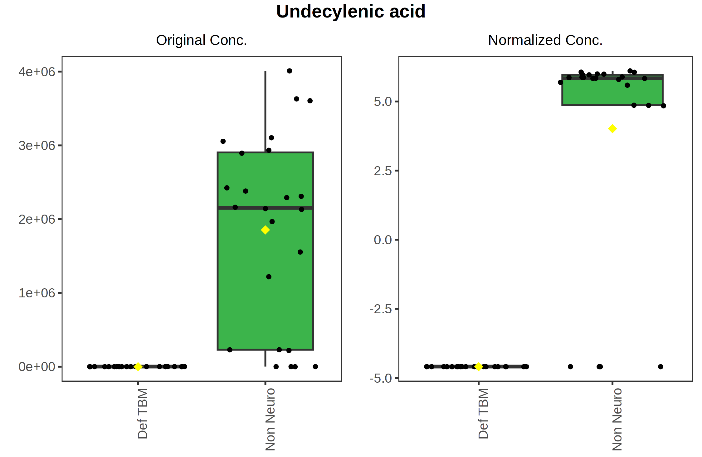 | Tartronic acid  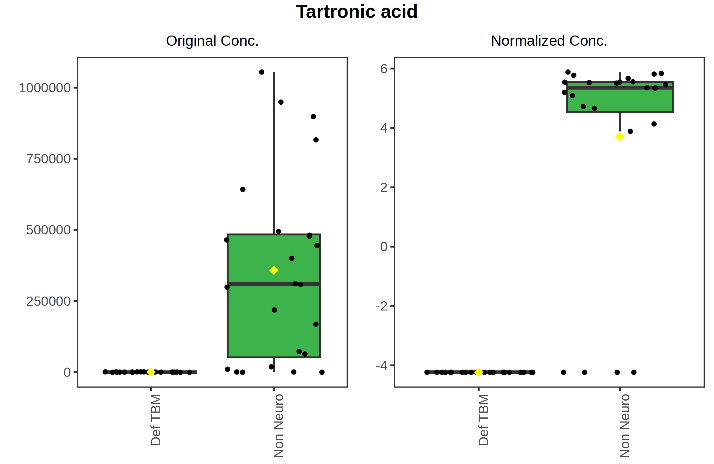 |
| --- | --- | --- |
| 3,5,5-trimethylhexan-1-ol  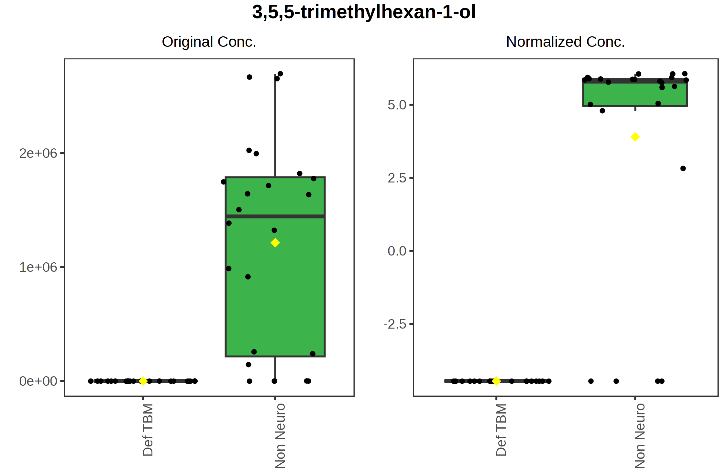 | Protein serine  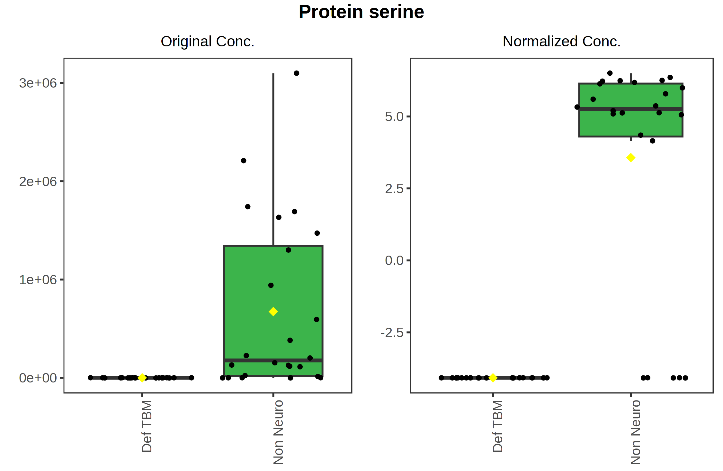 | Methyl galactoside  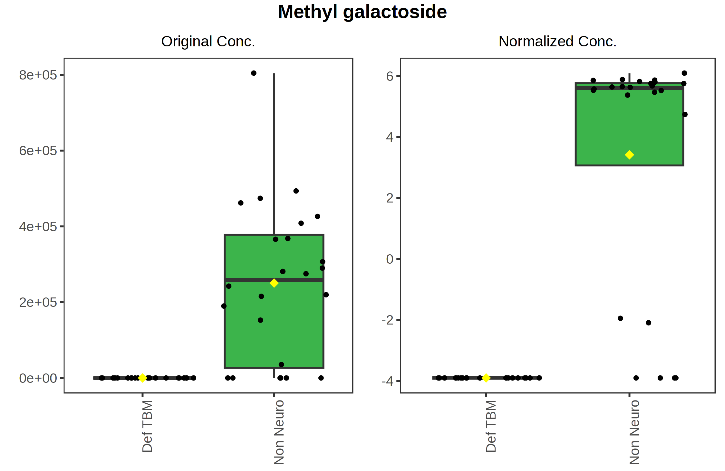 |
| Ribonolactone  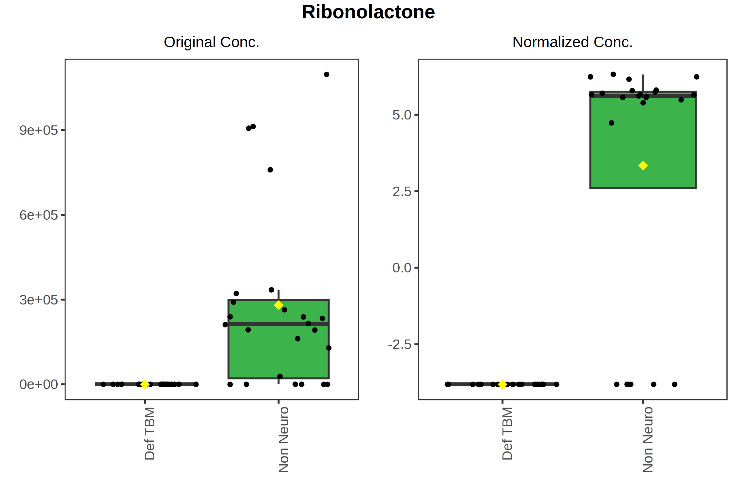 | 1-Butene, 1-butoxy-  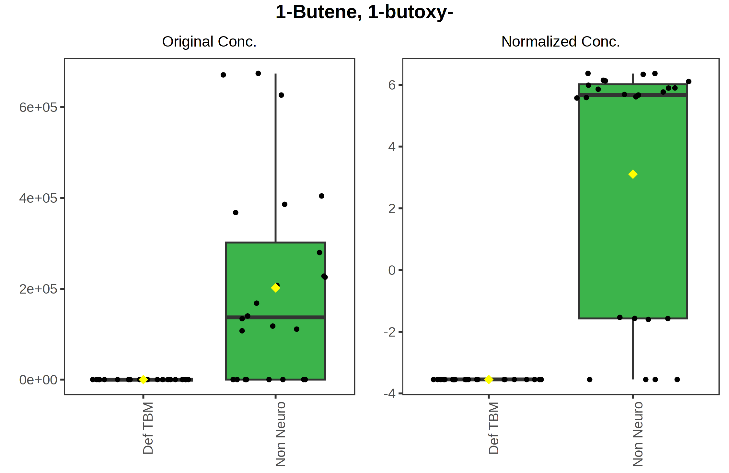 | 1-Pentene  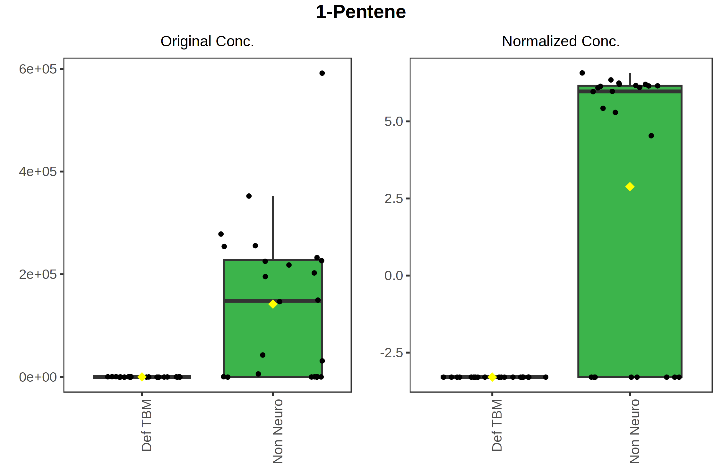 |
| Galactose  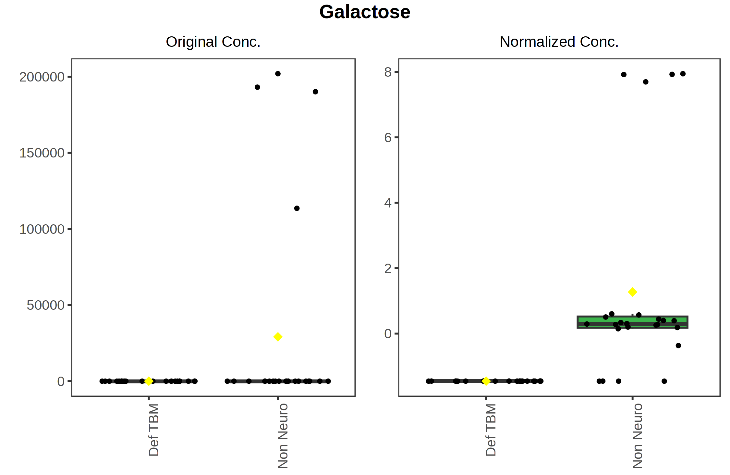 | Erythrono-1, 4-lactone  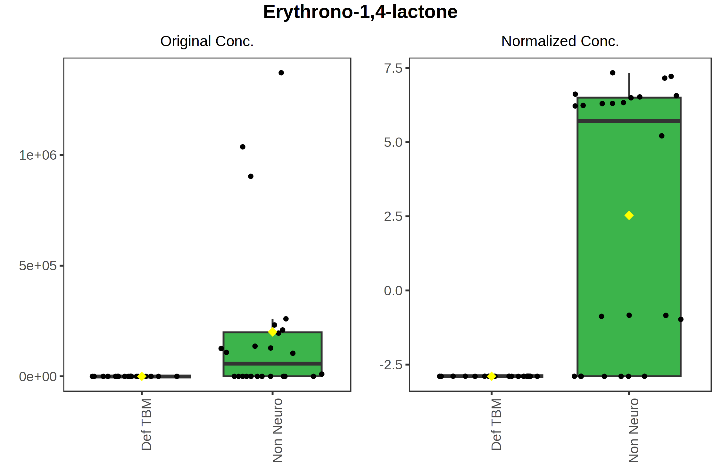 | 2-(2-Methoxyethoxy) acetic acid  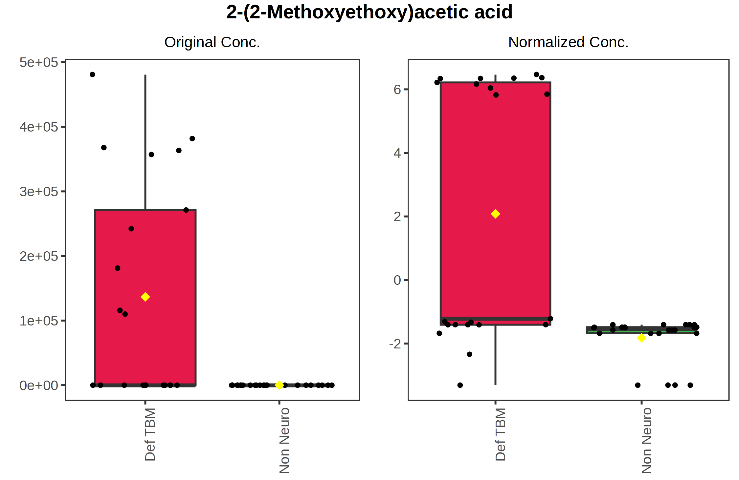 |
| 2-Ethylhexanol  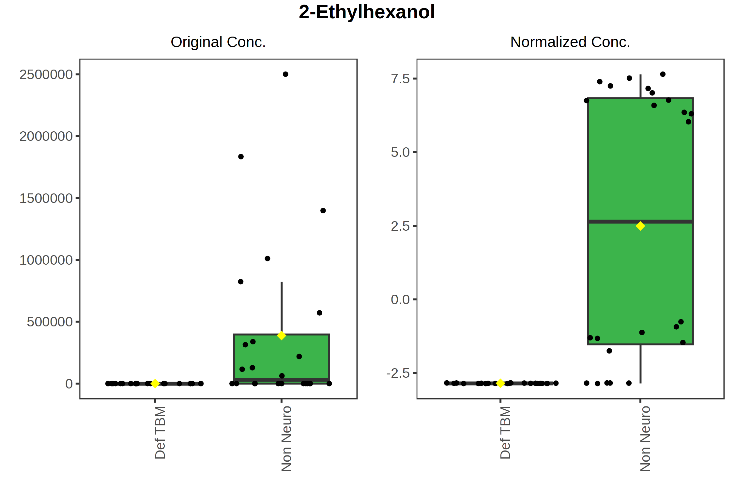 | Palmitic acid  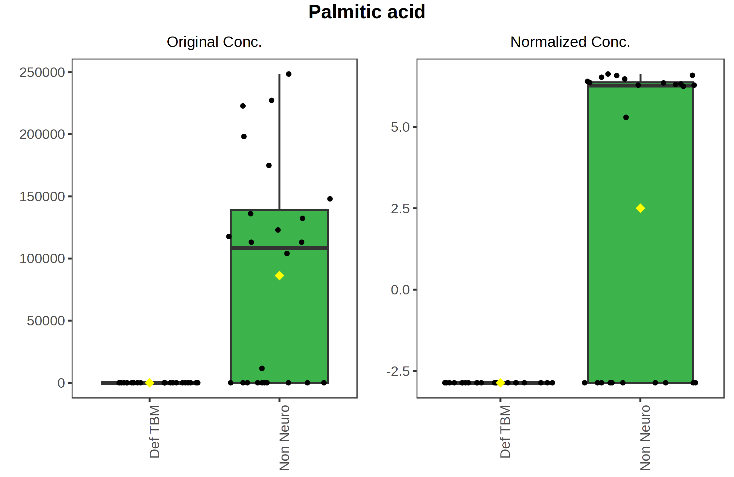 | 5-Ethyl-1,2,4-oxadiazol-3-amine  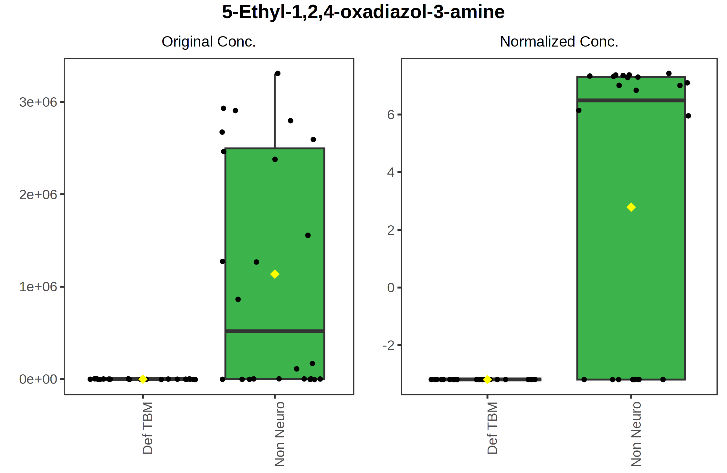 |
| 2-Ketoisocaproic acid  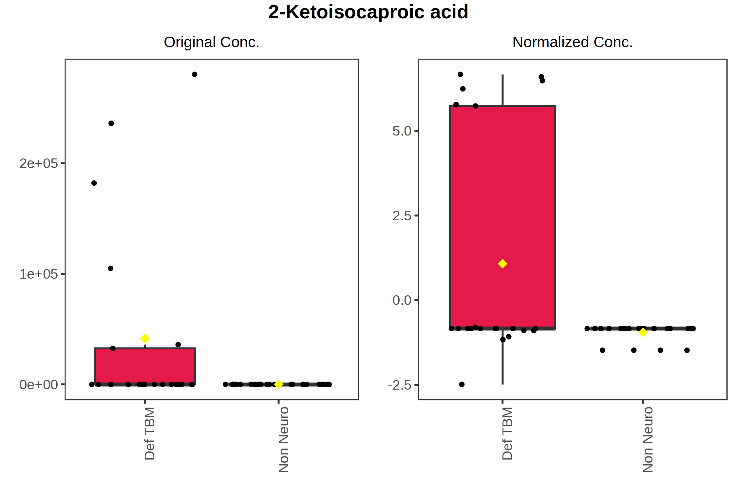 | | |

**Fig. S3** Box plots of the absolute concentrations (µmol/L) of the VIP metabolites identified using GCxGC-TOFMS

**Table S1:** Random Forest classification

|  | **Def TBM** | **Non-Neuro**  **Non-Meningitis** | **Class error** |
| --- | --- | --- | --- |
| **Def TBM** | 21.00 | 0.00 | 0.00 |
| **Non-Neuro**  **Non-Meningitis** | 3.00 | 21.00 | 0.12 |

**Table S2** Comparison of the 9 metabolites that are only present in the TBM groups, at various stages of TBM infection

| **TBM stage** | **1** | | **2a** | | **2b** | | **3** | |
| --- | --- | --- | --- | --- | --- | --- | --- | --- |
|  | **Prob TBM conc. (umol/L ± stdv)** | **Def TBM conc. (umol/L ± stdv)** | **Prob TBM conc. (umol/L ± stdv)** | **Def TBM conc. (umol/L ± stdv)** | **Prob TBM conc. (umol/L ± stdv)** | **Def TBM conc. (umol/L ± stdv)** | **Prob TBM conc. (umol/L ± stdv)** | **Def TBM conc. (umol/L ± stdv)** |
| **2-(2-Methoxyethoxy)acetic acid** | 0.00008±0.00011 | 0.00015±0.0002 | 0.00004±0.00007 | 0.00013±0.00016 | 0.0002±0.00028 | 0.00015±0.00015 | 0.00009±0.0001 | 0.00011±0.00017 |
| **2,3,4-Trihydroxybutanoic acid** | 0.00922±0.0039 | 0.01013±0.00704 | 0.00834±0.0014 | 0.00664±0.00255 | 0.00388±0.00047 | 0.0103±0.0103 | 0.00905±0.00766 | 0.00872±0.00375 |
| **2-Desoxy-pentos-3-ulose** | 0.00019±0.00005 | 0.00032±0.00024 | 0.00041±0.00033 | 0.00027±0.00007 | 0.00053±0.00032 | 0.00033±0.00033 | 0.00066±0.00073 | 0.00084±0.00095 |
| **2-Ketoisocaproic acid** | 0.00003±0.00005 | 0.00002±0.00004 | 0.00006±0.00011 | 0.00003±0.00005 | 0.00006±0.00008 | 0.0001±0.0001 | 0.00003±0.00005 | 0.00015±0.00013 |
| **5-Aminoimidazole** | 0.00036±0.00056 | 0.00103±0.00106 | 0.00003±0.00007 | 0.00074±0.00017 | 0.00152±0.00093 | 0.00024±0.00024 | 0.00056±0.00065 | 0.0004±0.00054 |
| **Arabinofuranose** | 0.00014±0.00017 | 0.00033±0.00019 | 0.00019±0.00018 | 0.00087±0.0015 | 0.0003±0.00005 | 0.00062±0.00062 | 0.00031±0.00022 | 0.00068±0.00059 |
| **Glyceric acid** | 0.00676±0.00204 | 0.00593±0.00204 | 0.0058±0.00148 | 0.00514±0.00272 | 0.00455±0.00232 | 0.0065±0.0065 | 0.00735±0.00409 | 0.00619±0.00199 |
| **L-Proline** | 0.0004±0.00078 | 0.00048±0.00118 | 0.0009±0.00103 | 0.00099±0.00089 | 0.00014±0.00008 | 0.00061±0.00061 | 0.00208±0.00219 | 0.004±0.00392 |
| **Sucrose** | 0.0011±0.00209 | 0.00045±0.00044 | 0.00142±0.00081 | 0.0033±0.00201 | 0.0014±0.00198 | 0.00166±0.00166 | 0.00482±0.00681 | 0.00165±0.00084 |
